# The Direction of Modern Therapies in Waldenström Macroglobulinaemia

**DOI:** 10.1111/jcmm.70987

**Published:** 2025-12-25

**Authors:** Stephen Blackmore, Sherine Elsawa, Omid Tavana

**Affiliations:** ^1^ College of Life Science and Agriculture, Department of Molecular, Cellular and Biomedical Sciences University of New Hampshire Durham New Hampshire USA; ^2^ Bioscience, Haematology R&D AstraZeneca Waltham Massachusetts USA

**Keywords:** cancer signalling, modern therapies, Waldenström macroglobulinaemia

## Abstract

Waldenström macroglobulinaemia (WM) is a rare lymphoplasmacytic disease that is hallmarked by B‐cell infiltration of the bone marrow, an overexpression of IgM class antibodies and an activating mutation of MYD88 (L265P). The therapeutic options for WM patients include a combination of Rituximab (anti‐CD20 monoclonal antibody) and chemotherapy, with newer treatments like proteasomal inhibitors and Bruton's Tyrosine Kinase (BTK) inhibitors showing high levels of success both as monotherapy and in combinations. To date, WM remains incurable. Understanding the basic physiology of WM and creating new and improved pre‐clinical models which better reflect the true physiology of WM will allow for the identification of novel therapeutic vulnerabilities and the ability to test these next generation therapies, both in a tumour intrinsic and extrinsic manner. In this review, we aim to provide a comprehensive summary of WM, focusing on the genetic mutations and signalling pathways driving disease progression. In addition, we highlight the current therapeutics and emerging clinical trials to provide novel insights to drive deep and durable responses.

## 1 Introduction

Waldenström macroglobulemia (WM) was first described by Dr. Jan Gosta Waldenström in 1944, as a lymphoplasmacytic disease with evidence of plasma cell differentiation [1, 2]. WM is a heterogeneous disease with varying amounts of small lymphocytes, plasmacytoid lymphocytes and plasma cells. WM can be diagnosed based on bone marrow infiltration by lymphoplasmacytic cells, with secondary consideration given to IgM levels, which vary greatly between patients [3, 4]. About 1500 new WM cases are diagnosed in the United States each year [5] which represents about 1%–2% of all haematological malignancies [6]. The average age of diagnosis is in the early 70s [5, 7].

The greatest risk factor for WM is progression from IgM‐monoclonal gammopathy of undetermined significance (MGUS), which carries a 46‐fold increase in likelihood of disease [8]. There are two other major risk factors that have been linked to WM. The first is a familial link; these patients are typically diagnosed with more aggressive disease and earlier onset [9]. The second is autoimmune diseases and familial history of autoimmune disease, which have some correlation with WM [10].

WM has a broad range of potential symptoms associated with tumour burden including blood hyperviscosity, which occurs in 35% of WM cases and is typically discovered due to fatigue, oronasal bleeding, retinal haemorrhage and/or an assortment of neurological disabilities [11, 12]. Mild neurological symptoms include headache, lightheadedness and fatigue, while the more severe symptoms include confusion, stroke and neurological deficits [13].

Cryoglobulinaemia (when monoclonal antibodies precipitate at temperatures < 37°C and redissolve when warmed) [14] occurs with high levels of IgM and associated blood hyperviscocity and requires urgent care [12]. Other WM‐associated ailments have unknown mechanisms such as Schnitzler's syndrome; a disease associated with skin lesions caused by monoclonal IgM [13, 15].

Schnitzler's syndrome along with MGUS causes patients to be closely monitored for potential transformation into WM [13, 15]. Waldenström Macroglobulinaemia B‐cells phenotypically express CD19, CD20, CD22 and CD24 while CD10, CD103 and CD11c are not expressed [12]. A small fraction (< 20%) were CD5 or CD23 positive [2, 12]. Soluble immunoglobulin (sIg) skews kappa over lambda 5:1 [12]. Despite this broad overarching phenotype, WM is a heterogeneous disease both at the genetic level (sub‐clonal) and cell‐state (differentiation state) levels.

## 2 Bone Marrow Microenvironment

**FIGURE 1:**
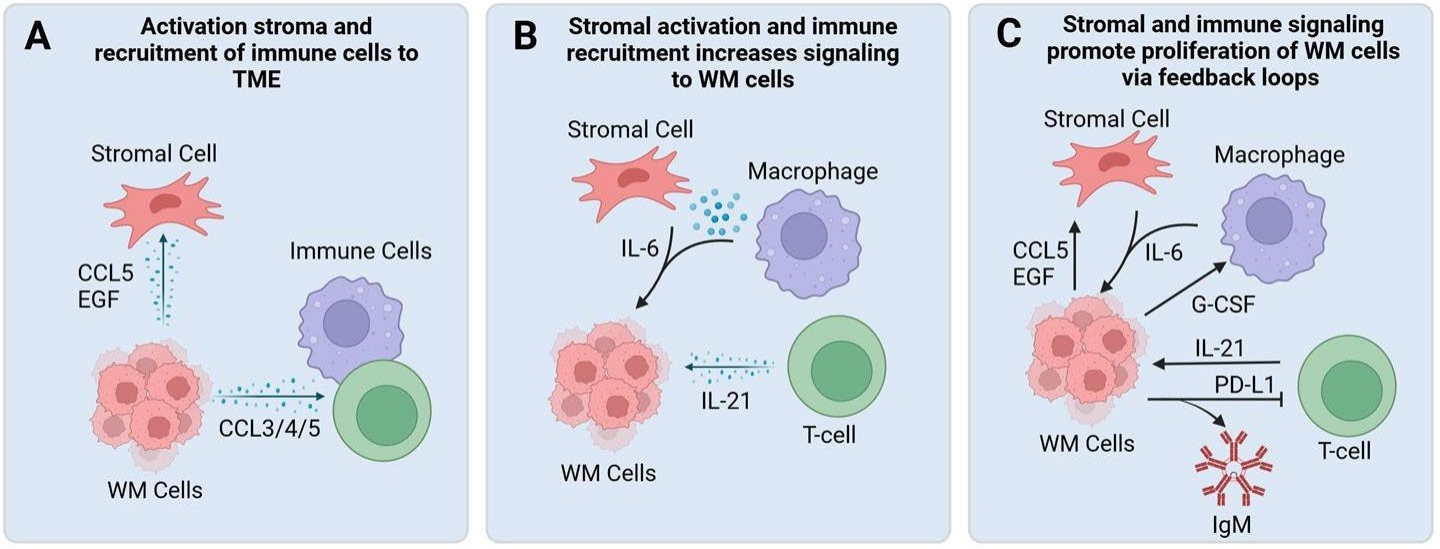
Overview of signalling crosstalk in the tumour microenvironment. WM cells recruit immune cells and signal bone marrow stromal cells and immune cells in feedback loops to create a TME conducive for tumour cell growth. Created using BioRender.com.

WM is a cancer of the bone marrow highly influenced by the tumour microenvironment, where all the bone marrow cells have a mechanism of crosstalk through secreted signalling molecules called cytokines and chemokines. These normal cells include immune cells, endothelial cells and bone marrow stromal cells (BMSC) such as chondrocytes, osteoblasts, fibroblasts, adipocytes and myocytes, which are all crucial for the growth and survival of WM (Figure 1). Co‐culturing WM cells with a stromal cell feeder layer has led to preclinical resistance of common therapies, suggesting stromal cells are necessary for tumour engraftment in immunocompromised mouse models [16, 17, 18, 19]. BMSCs are responsible for secretion of IL‐6, while T‐cells in the bone marrow have been shown to secrete IL‐21. Both have been linked to WM proliferation and increased secretion of IgM [20, 21]. Mast cells have also been found to be increased in about a quarter of patients [2]. CCL5 (RANTES) both from the WM cells and other cells in the microenvironment can act on BMSCs to secrete IL‐6 creating a positive feedback loop of proliferation for malignant B‐cells [22]. Several cytokines have also been shown to be aberrantly secreted in the serum of WM patients including upregulation of CCL5, G‐CSF, and soluble IL‐2 receptor and downregulation of IL‐8 and EGF [22]. While the role of CCL5 has been reported, the role of these other cytokines has yet to be investigated.

During WM pathogenesis there are several other immune cell populations with altered phenotypes beyond B‐cells. Myeloid cells are the most altered having a significantly diminished classical monocyte population and during the MGUS phase, an increased unique inflammatory phenotype expressing IL‐1β, CCL3, CCL4, IL‐6, NLRP3 and CXCL3 [23]. These cytokines are present in lower, but still elevated levels in WM patients. Natural Killer (NK) cells are also diminished in WM patients with increased exhaustion markers and a decreased interferon associated gene signature [23]. T‐cells had a drastically reduced naïve compartment and an increased CD8+granzyme+ compartment. This population also expressed the exhaustion marker TIGIT [23]. Like the NK cell group, both CD8+ and CD4+ T‐cells showed reduced expression of interferon genes [23]. Mast cells also have a well‐established role in WM promoting survival and tumour growth. Increased mast cell involvement in WM is linked to a shorter median overall survival and more aggressive phenotypes [24]. This survival benefit has been shown to signal through the CD40/CD40L axis [25, 26]. The interactions made between the cells of the TME and WM cells are just another potential vulnerability to be targeted by therapeutics. The communication between the TME and the cancer cells is rapidly becoming understood and targeting this communication network whether from a tumour intrinsic or extrinsic approach could be the key to new and improved therapies.

Another process commonly altered in cancer is angiogenesis. In the context of WM, angiogenesis is only elevated in about 30% of patients [27]. The angiogenesis associated cytokines, angiogenin, VEGF, VEGF‐A and bFGF are elevated in both MGUS and WM patients [28]. ANG‐1/ANG‐2 protein ratio decreased only in the WM patients and not in the MGUS patients [28]. Serum angiogenin levels were elevated during active disease and were lower in the control patients and patients with MGUS or in WM remission [28]. Only a weak correlation has been observed between bone marrow infiltration and an increase in vasculature, and no clinical correlation exists connecting angiogenesis directly to patient outcome [27].

Another factor of the microenvironment important to many cancers including WM is the oxygenation state. High oxygen in the microenvironment has been linked to higher levels of cell proliferation while hypoxic microenvironments have induced WM cells to extravasate into circulation and seed other bone marrow niches [29]. This occurs in a CXCR4/SDF‐1 chemotactic manner [29]. Hypoxia has been directly correlated with tumour burden, as marks of hypoxia increase with increased tumour burden in the bone marrow [29]. Hypoxia has also been linked to extravasation of WM tumour cells from the bone marrow into the circulation [29]. Clonal haematopoiesis (CH) is another phenomenon that has been linked to increased progression of IgM MGUS to WM, although it does not decrease overall survival [30]. In the context of WM, clonal haematopoiesis is when somatic mutation in haematopoietic stem cells leads to clonal expansion in the bone marrow. CH associated mutations have been observed in about 14% of WM patients [30]. Further understanding of the disease and TME: WM direct interaction may lead to the advent of novel therapies and a curative therapy with combinations.

## 3 Genetic Landscape

Waldenström Macroglobulinaemia is also characterised by several hallmark genetic mutations. More than 90% of WM patients are positive for a stimulatory Myeloid Differentiation primary response 88 (MYD88) mutation, specifically L265P. MYD88 is an adaptor protein downstream of Toll‐like receptor (TLR), IL‐1R and IL‐18R signalling [31]. It typically signals through Janus kinase/signal transducer and activator of transcription 3 (JAK/STAT3) and subsequently activates [32]. MYD88 L265P supports constitutive NF‐κB signalling by stimulating Bruton's Tyrosine Kinase (BTK) and Interleukin 1 Receptor Associated Kinase 1/4 (IRAK 1/4) [33, 34, 35]. MYD88 L265P is capable of regulating BTK activation, an activity not present in MYD88 WT cells [33].

Another prevalent mutation seen in WM is a C‐X‐C motif chemokine receptor 4 (CXCR4) Warts, Hypogammaglobulinaemia, Infections and Myelokathexis (WHIM)‐like mutation. About 25%–30% of patients harbour a mutation in the C‐terminal domain of CXCR4 [36, 37]. This mutation causes an impairment in CXCR4 internalisation in response to C‐X‐C motif chemokine ligand 12 (CXCL12) which enhances Protein Kinase B (AKT) and extracellular‐signal‐regulated kinase (ERK) activation [38, 39]. This mutation has also proven to be a resistance mechanism for the BTK inhibitor Ibrutinib [38, 40]. CXCR4 mutation strongly overlaps with MYD88 mutation, yet MYD88 mutation dominates as inhibition of MYD88 L265P removes all survival benefits bestowed by the CXCR4 WHIM‐like mutation [38, 41].

TP53 is a well‐known tumour suppressor whose function is lost across > 50% of human cancers [42, 43]. The protein, p53, is a nuclear transcription factor that plays important roles in cell cycle arrest, DNA damage repair and apoptosis [43, 44, 45]. TP53 abnormalities, including 17p deletions and TP53 mutations, are detected in approximately 5%–10% of patients who have not yet received treatment and in as many as 25%–30% of individuals with relapsed or refractory disease [46, 47, 48, 49]. Increasing evidence indicates that these genetic changes are linked to poor prognosis, with patients experiencing lower response rates, shorter progression‐free survival and decreased overall survival [46, 47, 48, 49].

Lysine Methyltransferase 2D (KMT2D; also referred to as MLL4) mutations additionally occur in a high percentage of WM patients. Around 20% of patients have a KMT2D mutation, although this mutation has been shown to be sub‐clonal in many of the patients [50]. KMT2D regulates gene transcription; in the context of a promoter, methylation marks added by KMT2D creating H3K4me3 activate gene transcription. KMT2D binds both promoters and enhancers and deposits H3K4 methylation to regulate gene transcription. To date, few reports have explored the functional consequence of different KMT2D mutations in WM, unlike those in FL and DLBCL where the frequency rates are 80% and 30%, respectively [51, 52, 53]. Better understanding of such epigenetic changes may lead to therapeutic vulnerabilities that may be exploited [54].

Inactivating AT‐rich interaction domain 1A (ARID1A) mutations occur in between 5% and 17% of patients [36, 50]. It is a switch/sucrose nonfermentable (SWI/SNF) family member responsible for chromatin remodelling/accessibility which controls gene expression. ARID1A mutations have been linked to higher levels of bone marrow involvement and more advanced disease. When the mutation occurs during the germinal centre reaction, it can lead to an increase in immature memory B‐cell fate that increases the likelihood of aggressive follicular lymphoma [55]. These follicular lymphomas have proven sensitive to therapeutic inhibition of SMARCA2/4 [55], which could be a targetable route forward in WM patients harbouring ARID1A mutants.

Other lesser common mutations identified in WM include: CD79B (7%), MYBBP1A (7%), PRMD1 (6%) and NOTCH2 (3%–5%) [36, 50]. All the previously mentioned mutations can alter WM cell signalling and modulate cellular pathways contributing to the pathogenesis.

There are also several cytogenetic abnormalities that occur in WM including trisomy 4 (tri4) (12%), tri18 (11%), del13q (11%), tri12 (7.5%) and del17p (7%) [49], the most common being the 6q chromosomal deletion (27%–50%) which is associated with poorer outcomes [47, 49]. Importantly, genes that regulate cell survival, BCR and NF‐κB signalling are lost in 6q deletions, although which are deleted varies depending on the size and location of the deletion. The following genes are most commonly lost: BCLAF1 (76%), TNFAIP3 (60%), HIVEP2, IBTK and FOXO3 (52%) [56]. 6q deletions are associated with the transition from MGUS to WM [57] as well as increasing in frequency with severity on the International Staging System prognostic index (prevalence of 6q‐ among patients in stages 1, 2 and 3 was 24%, 42% and 67% respectively) [58]. This scale has been tailored from multiple myeloma to WM by taking β2‐macroglobulin and albumin levels into account [59]. 6q deletion is also correlated with a corresponding inflammatory syndrome known as IWM (Inflammatory Waldenstrom Macroglobulinaemia) [60, 61]. This sub‐group represents about one third of WM patients and is characterised in Elessa et al. [60].

## 4 Signalling Pathways

**FIGURE 2:**
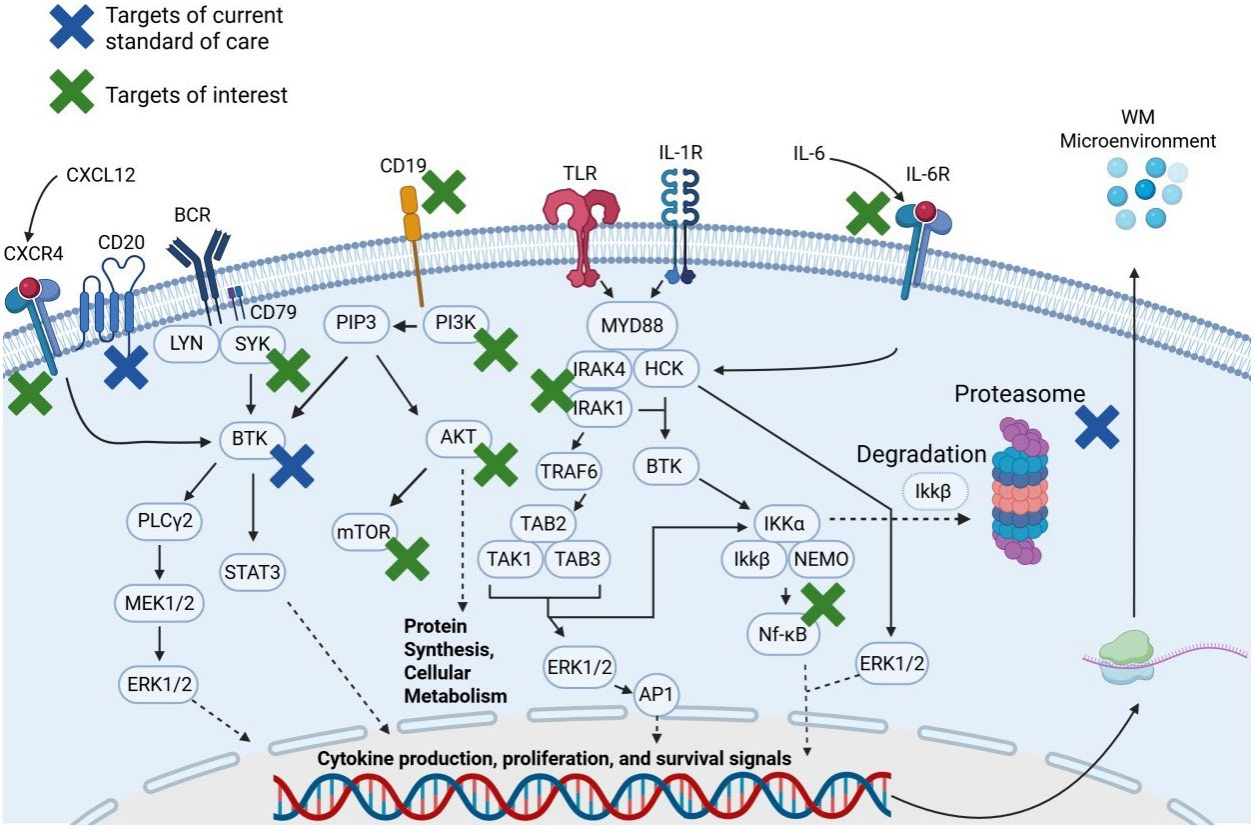
Important signalling cascades in Waldenström macroglobulinaemia. Overview of major signalling pathways involved in cytokine recognition and WM signalling as well as highlight of drug targets. Created using BioRender.com.

There are several signalling cascades that are vital to the survival and growth mechanisms of Waldenström Macroglobulemia (Figure 2). Typically the B‐cell receptor (BCR) signals down through CD79 and the kinases LYN and spleen associated tyrosine kinase (SYK) to stimulate BTK, but in WM, BTK can be activated downstream of MYD88 signalling and co‐stimulated by haematopoietic cell kinase (HCK) [33, 62]. MYD88 signalling is stimulated by upstream toll‐like receptors (TLR). This signalling cascade is constitutively activated in WM because of a mutated MYD88, which leads to constitutive activation of NF‐κB [33]. MYD88 can also signal through Interleukin 1 associated kinase 1/4 (IRAK1/4) to tumour necrosis factor receptor associated factor 6 (TRAF6) [63, 64, 65, 66]. TRAF6 then stimulates the transforming growth factor‐beta activated kinase 1 (MAP3K7) binding protein 2 (TAB2)/transforming growth factor‐β‐activated kinase 1 (TAK1)/transforming growth factor‐beta activated kinase 1 (MAP3K7) binding protein 3 (TAB3) complex which then either signals down to NF‐κB or can also participate in extracellular signal‐regulated kinase 1/2 (ERK1/2) activation [67]. ERK1/2 then activates the activator protein‐1 (AP‐1) transcription factor. BCR signalling is also hijacked by mutant MYD88 through the stimulation of SYK which signals down through STAT3 or to mammalian target of rapamycin (mTOR) via the protein kinase B (AKT) signalling cascade [68, 69].

Another signalling cascade important to WM is the IL‐6 receptor which feeds into the MYD88 pathway through HCK. HCK can then phosphorylate BTK and activate the AKT signalling pathway. The IL‐6 pathway downstream of the NF‐κB can be stimulated by TME cytokines or from autocrine signalling, which creates a positive feedback loop of survival and proliferation for WM cells [22].

CXCR4, another protein that is commonly mutated in WM leading to constitutive activation, also signals through BTK which then activates the phospholipase C‐gamma (PLCγ), diacylglycerol (DAG), protein kinase C (PKC) signalling cascade providing additional mechanisms to promote proliferation and survival in WM cells [41]. To further understand the functional consequence of these pathway alterations, in vitro models were derived from patients in an attempt to mimic the disease seen in patients. In this regard, WM models are limited to only three in vitro models isolated and characterised [70, 71, 72]; among these few models there are disputes over which of these cell lines best model WM disease.

## 5 Pre‐Clinical Models

WM has only a few preclinical models that can be utilised for research. These include MWCL‐1, BCWM.1, recently characterised BCWM.2 and RPCI‐WM1; these models have been phenotypically and genetically characterised by researchers at Mayo Clinic, the Dana Farber Cancer Institute and the Roswell Park Memorial Institute, respectively [70, 71, 72, 73]. Several groups have started to establish drug‐resistant versions of these cell lines particularly in relation to BTK inhibitor resistance. RPCI‐WM1 was derived from the lymph node of a 48‐year‐old female patient [70], MWCL‐1 was derived from the bone marrow of a 73‐year‐old male patient [71] and BCWM.1 was derived from the bone marrow of a female patient of undocumented age [72].

These cell lines also were established from patients at different stages of the disease. BCWM.2 was derived from the bone marrow of a 66‐year‐old male patient [73].

The BCWM.1 and BCWM.2 cell lines were derived from treatment‐naïve patients, while MWCL‐1 was derived from a patient who had three previous courses of chemotherapy [71, 72, 73]. RPCI‐WM1 was taken immediately postmortem from a patient that had undergone multiple therapeutic regimens over the course of 6 years. These regimens included fludarabine monotherapy, rituximab monotherapy, a combination of fludarabine and rituximab, a combination of bortezomib, pegylated liposomal doxorubicin and thalidomide, a combination of cyclophosphamide, dexamethasone and lenalidomide, and finally a combination of sildenafil, fludarabine and rituximab [70, 74]. Unsurprisingly, these cell lines react differently when treated with current therapies including ibrutinib and bortezomib [74]. Not only were these patients exposed to different therapies at the point of biopsy, but they have different mutation profiles. Interestingly, all four lines harbour a MYD88 mutation with BCWM.1, MWCL‐1 and RPCI‐WM1 having an L265P mutation, while BCWM.2 harbours a rare S243N mutation [73].

None of the lines contain CXCR4 mutations [75, 76]. Other mutations of note include a NOTCH2 mutation in BCWM.1 and a TP53 mutation in MWCL‐1 [75]. BCWM.2 also contains a heterozygous del6q which occurs in up to 70% of WM patients [73]. The vast differences between the origins of these cell lines underscore the need for additional preclinical models to fully parse out the mechanisms of WM and identify targets for future therapeutics that can work across a large portion of the WM population.

There are two transgenic mouse models that mimic parts of the WM phenotype by mutating MYD88 with a L252P mutation, the murine ortholog of the human L265P mutation [77, 78]. One established a genetic mutation in the MYD88 gene. This mutation led to elevated numbers of IgM producing plasma cells and a phenotype like MGUS [77]. The other model inserted the L265P mutation along with yellow fluorescent protein into CD19 positive cells [78]. This caused a similar increase in IgM producing B‐cell clones, but the cells localised to the spleen rather than the typical bone marrow niche seen in WM patients [78]. Despite both models providing useful tools to study facets of the disease, expensive and complicated humanised models remain the only way to study immune interactions between WM and the bone marrow TME, limiting preclinical research in WM.

## 6 Modern Therapeutic Regimens

Waldenström Macroglobulemia is currently uncurable, and treatments aim to slow progression of symptoms and prevent organ damage. When patients are asymptomatic, clinicians often will ‘watch and wait’, which involves routine observation without intervention until symptoms arise [79, 80, 81]. Outside of pharmaceutical interventions, plasma exchange and bone marrow transplant have been performed.

The first line therapeutic regimens currently prescribed to WM patients are BTK inhibitors and immunochemotherapy [82]. There are several considerations deciding which regimen patients are put on including CXCR4 mutational status, the frailty and age of the patients and even patient preference. CXCR4 mutation confers some level of BTK inhibitor resistance lowering the depth of response [82]. Immuno‐chemotherapy currently comes in two flavours, Bendamustine with Rituximab (BR) or Dexamethasone, Rituximab and Cyclophosphamide (DRC) [83]. The latter is considered mostly for older, more frail patients given its lower chance of adverse events given its lower rate of progression‐free survival [84]. There is a third immuno‐chemotherapy regimen showing promising data combining Bortezomib with DRC (B‐DRC) [85]. In a head‐to‐head trial of B‐DRC versus DRC, 24‐month progression‐free survival (PFS) was 80.6% for B‐DRC and 72.8% for DRC, which fails to reach statistical significance of improved PFS (*p* = 0.32) [85]. B‐DRC had a complete response or very good partial response in 17.2% versus 9.6% of patients in DRC [85]. Immuno‐chemotherapy regimens are also prescribed for patients that prefer a fixed duration therapy as BTK inhibitor treatment is a continuous course. More detail about the drug classes that make up these regimens is provided below.

### 6.1 BTK Inhibitors

BTK inhibitors Ibrutinib (first generation) and Zanubrutinib (second generation) have been approved for the treatment of WM. They bind covalently downstream of the B‐cell receptor at the protein Bruton Tyrosine Kinase (BTK). This prevents BTK from performing its kinase activity, which is important in several survival signalling pathways. Two other second generation BTK inhibitors have been approved for related B‐cell malignancies: Acalabrutinib and Tirabrutinib [86, 87]. Unfortunately, an acquired mutation to cysteine 481 on BTK will render the covalent inhibitors that bind to that residue inactive, as observed clinically in CLL [88, 89, 90].

### 6.2 Proteasomal Inhibitors

Proteasomal inhibitors inhibit the proteasome's ability to degrade proteins. This causes dysregulation in many pathways, but primarily NF‐κB and activates the endoplasmic reticulum (ER) stress response which is the main cause of apoptosis of WM cells. Inhibition of NF‐κB occurs because the inhibitory protein IκB, which is degraded by the proteasome, remains intact preventing pathway activation. Waldenström macroglobulinaemia cells are sensitive to this kind of pathway dysregulation. Proteasomal inhibitors like Bortezomib and Ixazomib have both shown efficacy in WM, both as a monotherapy and as a combination [91, 92, 93, 94, 95]. The dose limiting side effect for Bortezomib is peripheral neuropathy, with initial treatment regimens established as twice weekly [96, 97]. A head‐to‐head study of single weekly dosing versus biweekly dosing showed that this side effect can be limited with a single weekly dose, with similar efficacy in the context of multiple myeloma [97]. The negative effects of these drugs do not exacerbate the effects of other drugs, thereby making them an easy drug class to combine [93, 95].

### 6.3 Rituximab

Rituximab is an anti‐CD20 monoclonal antibody (mAb). This is the most commonly used first line therapy when treating WM patients and is predominately prescribed in combinations with other approved agents [98]. There has been a noted rise in serum immunoglobulin levels following initial treatment in about half of patients. This rise in IgM levels is referred to as ‘rituximab flare’, but this phenomenon subsides after about 4 months [99]. The rituximab plus cyclophosphamide, doxorubicin hydrochloride, vincristine (Oncovin) and prednisone regimen known as R‐CHOP has been the primary treatment for the past decade. Bendamustine, another chemotherapy, plus Rituximab has been shown to be a direct improvement over R‐CHOP [100] (Table 1).

**TABLE 1:** Efficacy results for past and present Waldenström macroglobulinaemia therapies in untreated patients.

Treatment class (Target)	Treatment	Dose/Regimen	Overall response rate (ORR)	Major response rate (MRR)	Progression free survival (PFS)	Overall survival	Symptoms/Notes
mAb (CD20)	Rituximab [99]	375 mg/m^2^ IV once weekly on day 1, weeks 1–4 and 17–20	48%	33%	20.3 months	90% at 3 years	IgM flare in 47% of cases
mAb (CD20), chemotherapy, chemotherapy, chemotherapy, corticosteroid	R‐CHOP (Rituximab, Cyclophosphamide, Doxorubicin, Vincristine, Prednisone) [155, 156, 157]	Rituximab 375 mg/m^2^ IV once weekly on day 1, cyclophosphamide 750 mg/m^2^ IV, doxorubicin 50 mg/m^2^ IV, vincristine 1.4 mg/m^2^ (max. 2.0 mg/day) IV, on day 1 and prednisone 100 mg/m^2^ oral on days 1–5. Treatment cycles were repeated every 3 weeks for a total of four to eight cycles	91%/96%	N/A	55.8% at 5 years	85.0% at 5 years	
Corticosteroid, mAb (CD20), chemotherapy	DRC (Dexamethasone, Rituximab, Cyclophosphamide) [158]	Six 21‐day courses of dexamethasone at 20 mg IV, rituximab IV 375 mg/m^2^ and oral cyclophosphamide 100 mg/m^2^ twice daily (days 1 to 5)	83%	74%	35 months	72.8%–81% at 2 years	IgM flare in 32% of patients
**Proteasome inhibitor, corticosteroid, mAb (CD20), chemotherapy**	**B‐DRC (Bortezomib, Dexamethasone, Rituximab, Cyclophosphamide) [** 85 **]**	**Bortezomib given SC at a dose of 1.6 mg/m** ^**2**^ **once daily on days 1, 8 and 15 for six cycles (28‐day interval) dexamethasone 20 mg oral once daily on day 1, rituximab 375 mg/m** ^**2**^ **IV once daily on day 1 of cycle 1 and 1400 mg SC once daily on day 1 of cycles 2–6, and cyclophosphamide 100 mg/m** ^**2**^ **× 2 oral days 1–5**	**N/A**	**80.6%**	**N/A**	**80% at 2 years**	**Modestly better major response when compared head–head against DRC in this trial. DRC used the same dosing strategy without the bortezomib**
**Chemotherapy, mAb (CD20)**	**BR (Bendamustine, Rituximab) [** 100, 157, 159 **]**	**Bendamustine 90 mg/m** ^**2**^ **IV days 1 and 2; rituximab 375 mg/m** ^**2**^ **IV on day 1; up to six 28‐day cycles**	**> 95%**	**> 95%**	**87% at 2 years/65.5% at 5 years**	**97.1% at 2 years/90.4% at 5 years/81.7% at 5 years**	**Neutropenia in 38% of patients, anaemia in 25%**
Proteasome inhibitor, mAb (CD20), corticosteroid	IRD (Ixazomib, Rituximab, Dexamethasone) [92, 160]	Ixazomib 4 mg PO days 1, 8, 15, dexamethasone 20 mg PO on days 1, 8 and 15, and rituximab 375 mg/m^2^ IV on day 1 (six 4‐week cyc les) followed by six 8‐week maintenance cycles	96%	77%	40 months	100% 3 years 4 months (9 had subsequent therapy) [92]	
Proteasome inhibitor, corticosteroid, mAb (CD20)	BDR Regimen 1 [93, 95]	Cycle 1: bortezomib 1.3 mg/m^2^ intravenously days 1, 4, 8 and 11 (21‐day cycle); cycles 2–5: bortezomib 1.6 mg/m^2^ intravenously days 1, 8, 15 and 22 every 35 days; cycles 2 and 5: dexamethasone 40 mg; rituximab intravenously 375 mg/m^2^ (total 8 infusions of rituximab); 5 cycles	85%	68%	43 months	66% at 8 years	
	Regimen 2 [91, 94]	Cycles 1–4: bortezomib intravenously 1.3 mg/m^2^; dexamethasone 40 mg on days 1, 4, 8 and 11; rituximab 375 mg/m^2^ day 11; cycles 5–8: as above, given 3 months apart; 4+4 cycles	96%	91%	57% at 5 years	95% at 5 years	
**BTK inhibitor**	**Ibrutinib [** 161, 162, 163 **]**	**420 mg/day, oral continuous**	**100%/100%/89%**	**83%/87%/67%**	**92% at 1 year 6 months/76% at 4 years**	**100% at 4 years**	
**BTK inhibitor, mAb (CD20)**	**Ibrutinib, Rituximab [** 99 **]**	**Ibrutinib 420 mg/day, oral, continuous, rituximab 375 mg/m** ^**2**^ **IV on day 1 of weeks 1–4 and 17–20**	**92%**	**72%**	**82% at 2 years 6 months**	**94% at 2 years 6 months**	
BTK inhibitor	Zanubrutinib [163, 164]	160 mg bid, oral, continuous	95%/100.0%	74%/87.5%	85% at 1 year 6 months/91.5% 2 years	84% at 1 year 6 months/100% 2 years	Only reporting treatment naïve cohorts
mAb (CD52)	Alemtuzumab [165]	30 mg dose given IV 3 times per week over 12 weeks	100%	80%	1 year 2.5 months for median time to progression	N/A	
mTOR inhibitor	Everolimus [166]	10 mg daily, oral, continuous	72.7%	60.6%	Median time to progression for major responders 2 years 9 months	N/A	Toxicity related discontinuation in 27% of patients

*Note:* Standard of care in bold.

### 6.4 Chemotherapies

Chemotherapies are still major players in WM combination therapies. Bendamustine and Cyclophosphamide are alkylating agents mentioned above in the combination therapies BR and DRC. They work by cross‐linking DNA base pairs, thereby interfering with DNA replication and repair mechanisms. Bendamustine also shares characteristics with purine analogs [101]. Purine analogs function by mimicking adenine and guanine during DNA and RNA synthesis and interfering with the processes. Cyclophoshamide is a prodrug which gets processed in the liver into 4‐hydroxycyclophosphamide and aldophosphamide [102]. Aldophosphamide then further breaks down into the active metabolite phosphoramide mustard [102].

Table 1 highlights the past and present treatment regimens tested on treatment naïve patients and their efficacy.

## 7 Therapeutic Perspectives Based on Emerging Mechanisms of Action Driven by Advances in WM Biology

The following therapeutics are an overview of the different drugs/classes that can potentially treat WM patients. Some of these therapies are approaching monotherapy approval, but many of them are being tested or will be used in combination with other agents to deepen responses.

Many of these combinations are with drugs previously mentioned like Rituximab, approved BTK inhibitors, or chemotherapies. The targets below that are logical extensions of current standards of care such as BTK degraders, next generation BTK inhibitors, or the BCL‐2 inhibitors are very far along in their development, while the antibody‐drug‐conjugates, bi‐specific antibodies and CAR‐T therapies are in their infancy. Below is a brief overview of the potential next generation WM therapies.

### 7.1 Antibodies

Antibodies are a rapidly growing field of therapeutics in WM patients. Currently, three monoclonal antibodies, Pembrolizumab (anti‐PD‐1) [103], Obinutuzumab (anti‐CD20) [104, 105] and Ulocoplumab (anti‐CXCR4) [106] are going through clinical trials, as well as two bispecific antibodies in PSB202 (anti‐CD20/CD37) [107] and XmAb13676 (anti‐CD20/CD3) [108]. Anti‐IL‐6 antibodies were intriguing given the target's involvement in tumour growth, survival and IgM production [20], but when tested in WM patients, they did not provide enough efficacy to enter standard of care [109]. All these antibodies either target highly expressed B‐cell surface markers (CD20, CD37), major altered pathways in WM (CXCR4), or are targeted at stimulating the surrounding immune system (CD3, PD‐1, IL‐6).

### 7.2 Small Molecule Inhibitors

Small molecule inhibitors are the most numerous classes of drugs targeting WM involving both monotherapies as well as being integral to most combination regimens. There are several major pathways that are being targeted with these inhibitors. The first, previously mentioned above, are BTK inhibitors. Acalabrutinib is not yet approved for WM, but is undergoing clinical trials. Acalabrutinib [86, 110], like Zanubrutinib and Ibrutinib, is a covalent BTK inhibitor. Pirtobrutinib [111, 112] and AS‐1763 are non‐covalent inhibitors [113].

Pirtobrutinib does not share this binding site or binding mechanism and therefore is effective in patients who have acquired this mutation after undergoing covalent BTK inhibitor treatment [88]. There is still potential for acquired resistance using Pirtobrutinib, although the resistance mechanisms may differ, allowing for the potential of sequencing different BTK agents. It is assumed that these advantages could be extrapolated to AS‐1763 as well as other BTK inhibitors that do not share the covalent binding mechanism.

### 7.3 BTK PROTACs

BTK PROTACs (proteolysis targeting chimeras) bind to BTK and hijack an E3 ligase into proximity of BTK, forcing BTK ubiquitination and subsequent degradation. There are currently three BTK degraders in the clinic for WM: BGB‐16673 [114] and NX‐5948, which are BTK specific degraders [115] and NX‐2127, which is a BTK/IKZF3 dual degrader [116].

Another WM therapeutic vulnerability is through targeting the PI3K/AKT pathway. This can be done by using Capivasertib and directly targeting AKT or by targeting Phosphoinositol‐3‐Kinase (PI3K) using BR101801 (PI3Ky/d) [117] or Idelalisib (PI3Kd) [105]. The AKT signalling pathway can signal downstream of HCK, which in the case of WM feeds into the dysregulated function of MYD88 signalling [118, 119]. This pathway is most well known for being a metabolic, growth and proliferation regulator. In WM, overactivation of this pathway has been identified as a potential resistance mechanism for the previously mentioned BTK inhibitor Ibrutinib [120] identifying a potential vulnerability for this resistant patient population or a combination approach in an earlier disease setting to prevent this resistance.

A third group of inhibitors directly induces apoptosis by targeting BCL‐2 and includes Venetoclax [111, 112, 121], APG‐2575 [122] and BP1002, a L‐Bcl‐2 anti‐sense oligonucleotide [123]. Venetoclax has already proven to be an effective therapy in WM in the relapse/refractory space, including those with previous BTK inhibitor treatment [124]. This shows the future promise of not only Venetoclax but all BH3 mimetics. BH3 mimetics are a group of cell death agents that bind to the BH3‐only protein (initiators of apoptosis) binding sites on BCL‐2 family members, inducing their apoptotic function [125]. Th BCL‐2 is an anti‐apoptotic protein with related family members Bcl‐xL, Mcl‐1, Bcl‐w and A1/Bfl‐1 [126]. Inhibition of the anti‐apoptotic family of proteins leads to an imbalance of the pro and anti‐apoptotic proteins leading to cell death. WM might be sensitive to this mechanism of inhibition due to their low expression levels of both pro and anti‐apoptotic proteins on the mitochondrial membrane [126].

Immunomodulators (IMiDs) are another subclass of small molecule inhibitors. This class includes Pomalidomide [127] and Lenalidomide [104, 128] and works by binding and hijacking Cereblon [129]. Cereblon is a substrate receptor for the Cullin‐4‐RING Ubiquitin Ligase (CLR4) [130]. These drugs act as a molecular glue, bridging CRBN‐CLR4 to neoantigens like Ikaros and Aiolos, driving their degradation [131, 132]. The degradation of these proteins can increase dendritic cell: effector T‐cell co‐stimulation in the bone marrow microenvironment, stimulate cytokine secretion, while having a direct tumour intrinsic function in multiple myeloma cells [133, 134]. The details of these mechanisms are well spelled out in a review by Cippitelli et al. [135]. IMiDs have been predominantly deployed as part of a combination strategy.

Compounds targeting the NF‐κB pathway are also of interest due to WM's tonic MYD88 signalling. A compound currently in the clinic for WM is SGR‐1505, which is a Mucosa‐associated lymphoid tissue lymphoma translocation protein 1 (MALT1) inhibitor [136]. MALT1 is a proteolytic enzyme that has shown involvement in NF‐κB activation. Inhibition of MALT1 has led to decreases in NF‐κB dependent cell survival and proliferation gene signatures in ABC‐DLBCL [137]. There are also compounds that have not entered the clinic for WM but are being tested for other indications that target this pathway and should be considered potential WM therapeutics. Those include drugs targeting MYD88 itself [138], IRAK4 (emavusertib) [139], or a combination of IRAK1/4 [140, 141]. Targeting of this major signalling pathway has shown success in pre‐clinical WM models [142, 143].

### 7.4 Cell Therapies

Cell therapies are a relatively new form of therapeutic intervention. There is currently Brexucabtagene Autoleucel (CD19‐targeted CarT) [144], MB‐106 (CD20‐targeted CarT) [145], the combination of CLBR001/SW1019 [146] which are a CarT and humanised antibody switch to control activation of the CarT and NKX019 (NKG2D‐targeted CarT) [147]. Most of these are classical CarT constructs, but the CLBR001/SW1019 is a ‘universal’ CarT with a targeting ‘switch’ mechanism in SW1019 [148]. SW1019 targets the specific tumour antigen and presents a peptide that binds to CLBR001 cells creating a link between the tumour cells and the CarT allowing for tumour cell killing [148]. Each treatment is inactive without the other [148].

### 7.5 Antibody Drug Conjugates

Antibody drug conjugates (ADC) involve a targeting portion and a warhead that is being specifically delivered to cells that possess the targeted marker. There are currently two ADC therapies going through clinical trials for WM. Loncastuximab teserine [149] is an anti‐CD19 targeted antibody drug conjugate (ADC) with a pyrrolobenzodiazepine (PBD) warhead. PBD is a DNA alkylating agent. Iopofosine [150] uses a small molecule phospholipid ether to target the delivery of iodine‐131. Phospholipid targeting works by having a high affinity for lipid rafts.

Once bound, these phospholipids flip across the membrane, resulting in delivery of the warhead directly into the cell. This compound has had success in relapse‐refractory WM patients detailed in the CLOVER‐WaM trial [151, 152].

## 8 Future Directions

Despite progressively better outcomes produced by modern therapies, none have proven to be curative for WM patients. There are several fields of study that would benefit this patient population. One of these directions is the identification, creation and comparison of additional pre‐clinical WM models. As mentioned previously, the cell line models that exist for WM come from radically different therapeutic and circumstantial backgrounds [70, 71, 72, 73]. Without additional models, teasing out why these cell lines might respond to certain therapies while others do not is difficult. A limited number of cell lines makes identifying and deeply understanding pre‐clinical drug responses and building confidence in driving targeted therapies in WM difficult. There are no established syngeneic models of WM, no publicly available patient‐derived xenograft (PDX) models and only a single attempt at a genetically engineered mouse model has cleared peer review, which only partially mimics WM [153]. This means the best mouse models available to pre‐clinical researchers are immuno‐compromised animals in an indication that has well‐established crosstalk with the immune microenvironment [20, 21, 22, 23, 24, 25, 26]. This only allows for the testing of tumour intrinsic agents that can have activity without any other cell type, or it requires the use of expensive and complicated humanised mouse models. Adding to these preclinical models would make identifying and validating targets as well as testing the basic physiology of the disease easier and more reliable.

One strategy to compensate for the lack of WM models is to compare the activity of drugs in lymphoma models sharing similar mutations to WM, like those of MYD88. Even if preclinical activity is seen in these lymphomas, WM patients may respond differently than predicted. WM research would benefit from taking a broader look at how current and developing therapeutics alter the behaviour of relevant cell types in the microenvironment. Many therapeutics and combination regimens could be affecting the tumour microenvironment as well as directly acting on the tumour. This is directly addressed with immuno‐oncology drugs, but even drugs such as BTK inhibitors which have previously focused on B‐cell effects in WM can have broader ramifications and potential efficacy outside of the on‐target tumour activity [154]. BTK is present in more than just B‐cells, and ongoing work is elucidating the consequences of these ‘on‐target off‐tumour’ effects. Together, current model development efforts, new therapeutic options, and a better understanding of current therapies can help to manage WM.

## Author Contributions

**Stephen Blackmore:** conceptualization (equal), writing – original draft (equal). **Sherine Elsawa:** conceptualization (equal), writing – original draft (equal). **Omid Tavana:** conceptualization (lead), supervision (lead), writing – original draft (equal).

## Conflicts of Interest

S.B. and O.T. are employees and shareholders of AstraZeneca.

## Data Availability

Data sharing not applicable to this article as no datasets were generated or analysed during the current study.
